# Assessing Autism Spectrum Disorder in People with Sensory Impairments Combined with Intellectual Disabilities

**DOI:** 10.1007/s10882-018-9597-x

**Published:** 2018-04-24

**Authors:** Gitta de Vaan, Mathijs P. J. Vervloed, Nienke C. Peters-Scheffer, Tiejo van Gent, Harry Knoors, Ludo Verhoeven

**Affiliations:** 1 Behavioural Science Institute, Radboud University, PO Box 9104, 6500 HE Nijmegen, The Netherlands; 2 Driestroom, PO Box 139, 6660 HC Elst, The Netherlands; 3 Royal Dutch Kentalis, Theerestraat 42, 5271 GD Sint-Michielsgestel, The Netherlands; 4 Royal Dutch Kentalis, Kentalis Academy, Theerestraat 42, 5271 GD Sint-Michielsgestel, The Netherlands

**Keywords:** Autism spectrum disorder, Intellectual disabilities, Multiple disabilities, Sensory impairments, Assessment, Observation

## Abstract

People with sensory impairments combined with intellectual disabilities show behaviours that are similar to Autism Spectrum Disorder (ASD). The instrument Observation of Autism in people with Sensory and Intellectual Disabilities (OASID) was developed to diagnose ASD in this target group. The current study focuses on the psychometric properties of OASID. Sixty individuals with intellectual disabilities in combination with visual impairments and/or deafblindness participated in this study. The OASID assessment was administered and rated by three independent observers. By means of expert consensus cut-off scores for OASID were created. To determine the concurrent validity OASID was compared with the Pervasive Developmental Disorder for People with Mental Retardation (PDD-MRS) and the Childhood Autism Rating Scale second edition (CARS-2). The intra-rater reliability, the inter-rater reliability, internal consistency and concurrent validity of OASID were good to excellent. Cut-off scores were established based on criteria from the DSM-5. OASID was able to differentiate between four severity levels of ASD.

## Introduction

People who have intellectual disabilities combined with visual impairments or deafblindness show impairments that may also occur in autism spectrum disorder (ASD) (de Vaan et al. 2013; Hoevenaars-van den Boom et al. 2009; van Gent 2012). ASD is characterised by impairments in communication and social interaction, repetitive and stereotyped behaviour and resistance to change (American Psychiatric Association 2013). These impairments, however, are not exclusive to ASD. Some of these impairments are, for example, also seen in people with social communication disorders (American Psychiatric Association 2013), insecure attachment styles (Zeanah et al. 2005; Rutgers et al. 2004), combined sensory and intellectual disabilities (de Vaan et al. 2016b) and even in typically developing children (Frith 2003). In order to diagnose and classify atypical behaviour correctly it is important to establish what causes a person’s behaviour. Determining the aetiology of behaviour is required for choosing an appropriate intervention strategy. To this end the diagnostic instrument ‘Observation of Autism in people with Sensory and Intellectual Disabilities’ (OASID) was developed in a pilot study (de Vaan et al. 2016b). The aim of OASID is to correctly diagnose the presence of ASD in individuals with a moderate to profound intellectual disability, combined with a visual impairment or deafblindness. The pilot study showed good inter-observer agreement, excellent internal consistency and adequate content and construct validity (de Vaan et al. 2016b). The pilot, however, studied only 18 people making it hard to calculate cut-off scores. The current paper focuses on improving the calculations of the psychometric properties: firstly by including more participants, secondly by adding an extra instrument for calculating the construct validity, and thirdly by proposing cut-off scores.

It is difficult to correctly interpret ASD symptoms in persons with combined sensory and intellectual disabilities because symptoms are often not unique to ASD alone. Persons with hearing loss and visual impairments can show drawbacks and peculiarities in language and communication (Knoors and Vervloed 2011; Wolters et al. 2011; Cass 1998; Dale et al. 2014; Fraiberg 1977; Gense and Gense 2005) and a delay in the development of Theory of Mind (Peterson and Siegal 2000; Peterson et al. 2000). These impairments are similar to some diagnostic criteria for ASD that were mentioned earlier. In addition, people with visual impairments also show frequently stereotyped behaviours (Tröster et al. 1991). Finally, an intellectual disability can also be the cause of behaviours that are often found in ASD, with symptom overlap becoming larger as the intellectual disability is more severe (Matson et al. 2008; Matson and Shoemaker 2009). The combination of sensory and intellectual disabilities make it even more likely that these people show behaviour patterns often seen in ASD (Carvill 2001). Especially in the domains of social and communicative development and daily living skills people with both sensory and intellectual disabilities show many typical features of ASD (Dalby et al. 2009; Evenhuis et al. 2009; Hoevenaars-van den Boom et al. 2009; Munde and Vlaskamp 2014). Examples of these behaviours include: stereotyped movements, such as hand waving and body rocking (Gense and Gense 2005; Medeiros et al. 2014), lack of reciprocity in social interaction (Dale et al. 2014), poor use of language for social purposes and awkward pragmatic language use (Tadić et al. 2010). Prevalence of ASD in people with sensory or intellectual disabilities is much higher than the 1–2% normally found in the total population (CDC 2017). Almost half (about 56%) of children identified with ASD have below average intellectual ability (CDC 2017). According to Jure et al. (2016) ASD is 30 times more frequent in children who are blind than in the sighted population and Cass (1998) mentioned that about one third of totally blind children show symptoms of ASD. ASD is also common in deaf children although this seems to be related more to comorbid intellectual disabilities than to deafness per se (Jure et al. 1991).

In addition to the overlap in behaviour, there is a lack of diagnostic and assessment instruments that are suitable for people with the above mentioned disabilities (Bodsworth et al. 2011; Hoevenaars-van den Boom et al. 2009; Jure et al. 1991; de Vaan et al. 2016a). In most cases diagnostic instruments do not have norms for people with combined sensory and intellectual disabilities; see for instance the manuals of the ADOS (Lord et al. 1999), ADI-R (Rutter et al. 2003) and a validity study on the Autism Behavior Checklist (Krug et al. 1980). Furthermore, testing procedures do not often take sensory impairments into account (Tobin and Hill 2011). Sometimes researchers and clinicians adjust diagnostic instruments for people with sensory impairments, but these adjustments are usually not validated or are found inappropriate by Williams et al. (2014). Because of the high prevalence of symptoms of ASD in people with multiple disabilities, even persons without ASD can score above the ASD cut-off on diagnostic instruments (Dammeyer 2014). For these reasons, there is an urgent need for the development of new assessment procedures for people with sensor or intellectual disabilities, or a combination of both (Nakken and Vlaskamp 2007; Tobin 1994).

ASD or ASD-related features among congenitally blind, deaf and deafblind children have been reported in clinical studies (see Dammeyer 2014). The main problem with these studies is the use of instruments that are invalid for these populations (de Vaan et al. 2016a). Despite the difficulties in diagnosing ASD in people with combined sensory and intellectual disabilities, research has shown it is possible to differentiate people with and without ASD (Dammeyer 2014; de Vaan et al. 2016b; Hoevenaars-van den Boom et al. 2009). The development of the OASID fits into this line of research. OASID was developed to help to diagnose ASD in people with combined sensory and intellectual disabilities. OASID is a semi-structured observational instrument for diagnosing ASD in this target population. In an earlier study with 18 participants, OASID proved to be a reliable and valid diagnostic instrument (de Vaan et al. 2016b). Given the small size of the study sample, replication with a larger group of participants was deemed necessary. Note that OASID is not designed to replace a full psychological assessment, including interviews, patient history, observations and testing. OASID is intended as a useful addition to existing assessment methods.

In the current study, people are included with moderate to profound intellectual disabilities, combined with visual impairment or deafblindness. Though the term deafblindness might imply complete lack of both sight and hearing, it can also be defined as any given combination of visual and auditory impairments (Dammeyer 2012; Ask Larsen and Damen 2014). The latter definition was used in this study. This paper consists of a description of some psychometric properties of OASID and a proposed heuristic to classify people with ASD that is in line with the current classification of ASD in the DSM-5 (American Psychiatric Association 2013).

## Methods

### Participants

Participants were 60 individuals (42 male, 18 female) with ages ranging between 6 and 55 (*M* = 31.6, *SD* = 14.9), 17 of which were children (18 or younger). The broad age range was used to ensure ample potential participants. Earlier studies have indicated that in persons with intellectual disabilities, especially when over 65, the prevalence of dementia is significantly higher than in the typical population (Cooper 1997; Strydom et al. 2009). To prevent possible interference of behaviours that are due to dementia or old age, a conservative maximum age, 60 instead of 65, was used as exclusion criterion. Participants had moderate (*n* = 11), severe (*n* = 24) or profound (*n* = 25) intellectual disabilities. A total of 34 participants used verbal language to communicate, ranging from a few words to full sentences, whereas two used sign language, two used tactile sign language, and 22 used no language or communication system at all. OASID administrators spoke verbally to participants or with a combination of verbal and sign language. All participants had a visual impairment, 30 of them were blind with or without light perception. Of all participants 16 were deafblind. Information regarding intellectual disability and sensory impairments was collected from the records of the participants and were established in the past by licenced psychologists, physicians, ophthalmologists and audiologists independent of the current study. According to the medical records the aetiologies of the disabilities were: prematurity (*n* = 8), brain damage (*n* = 6), congenital rubella syndrome (*n* = 5), Down syndrome (*n* = 5), Leber’s amaurosis (*n* = 4), Goldenhar syndrome (*n* = 2), Angelman syndrome (*n* = 1), consanguineous parents (*n* = 1), Bardet-Biedl syndrome (*n* = 1), other birth deficits (*n* = 11). For 16 participants the aetiology was unreported or unknown. Participants were recruited in collaboration with four residential institutions and three schools for people with intellectual and sensory disabilities throughout the Netherlands. To maintain privacy and anonymity until participation was confirmed, recruitment was performed entirely by institutional staff, and thus not the research team. The selection criteria were: a moderate to profound intellectual disability combined with a visual impairment or blindness according to the criteria of the ICD-10 (World Health Organization 2016), or deafblindness, which was defined as any combination of a visual and auditory impairment.

Within our study, no subgroups were made based on age, level of intellectual disability or level of sensory impairments. This was done since, with the current number of participants and the number of potential subgroups, the number of participants per group would be very small, resulting in limited statistical power.

### Materials

#### Observation of Autism in People with Sensory and Intellectual Disabilities

OASID is a semi-structured observational assessment for ASD in people with combined sensory and intellectual disabilities (de Vaan et al. 2016b). An experimenter conducted five tasks with each participant in a playful manner, adjusting communication and play level to their abilities and impairments. For example, one task consisted of a puzzle with four different degrees of difficulty that could be adjusted to the participant’s cognitive and motor abilities. Communication about the puzzle was achieved through spoken language, sign language, tactile sign language or by simply presenting puzzle pieces, depending on the participant’s communication style.

The play session was recorded on video and scored offline to a 40-item checklist. Items had three possible scores, ranging from 0 to 2, reflecting absent, intermediate or full presentation of features of autism.

All items were accommodated to the participant’s level and type of impairment. For example, it was asked if the participant responded to initiations for contact or sought the researcher’s attention, whether by eye contact or alternative means if the participant was blind. Additional examples of seeking contact in an alternative way were given, for example ‘taking the researcher’s hand’ or ‘talking to the researcher’.

Scores on individual items were added to obtain a total score and a score on two scales, namely: ‘Social Behaviour and Communication’ and ‘Repetitive and Stereotyped Behaviour’. These two scales were based on the domains of ASD described in the DSM-5 (American Psychiatric Association 2013). In line with the criteria described in the DSM-5, seven subscales were defined. The first three subscales are part of the first scale, namely ‘reciprocity’, ‘non-verbal communication’ and ‘relationships’. The following four subscales are part of the second scale ‘stereotyped and repetitive behaviours’, ‘insistence on sameness’, ‘restricted and fixated interests’ and ‘hyper- or hypo reactivity to sensory input’. High scores indicated more ASD typical behaviours. These diagnostic criteria were transformed into testable items based on existing items in the O-ADB (Hoevenaars-van den Boom et al. 2009) and the ADOS (Lord et al. 1999), in addition to expert experiences and observations of how diagnostic criteria may express themselves in the current target populations. An earlier study found the reliability and validity of OASID to be good (de Vaan et al. 2016b). The inter-rater reliability was demonstrated by a weighted kappa of 0.75 and an intraclass correlation coefficient of 0.69. The internal consistency showed a Cronbach’s alpha of .94 for both scales. Construct validity in the pilot study was established by looking at divergent and convergent validity. A lack of a significant correlation with the list for disturbed attachment was found, *r* =. 46, *p* = .57 and a positive correlation with the PDD-MRS (as described below), *r* = .40, *p* = .049 respectively.

#### Pervasive Developmental Disorders in Mental Retardation Scale

In the current study the original Dutch version of the Pervasive Developmental Disorders in Mental Retardation Scale (PDD-MRS; Kraijer and de Bildt 2005; Kraijer 1999) was used to determine concurrent validity. The PDD-MRS is a 12-item questionnaire designed to diagnose ASD or Pervasive Developmental Disorders (PDD) in people with intellectual disabilities. Questions can be answered with a positive or a negative score; all the positive scores were counted and weighed to result in a total score. Scores of 10 and above indicated ASD, scores of 6 and below indicated no ASD; scores that were in between gave uncertain results. The PDD-MRS is found to have good inter-rater reliability, test-retest reliability and internal consistency (Evers et al. 2010; Meadows 2007). Content validity was also good, criterion validity was sufficient, and sensitivity and specificity were good (Evers et al. 2010; Kraijer 1999; Meadows 2007; O'Brien et al. 2001).

The PDD-MRS was chosen as a measure of concurrent validity within this study because it is one of the few instruments that was specifically designed for ASD in people with intellectual disabilities (Kraijer and de Bildt 2005). Additionally, it is an originally Dutch measurement and its interpretations are based on a Dutch sample, increasing the validity for use in our Dutch sample.

#### Childhood Autism Rating Scale 2

The Childhood Autism Rating Scale, Second Edition (CARS2; Schopler et al. 2010) was used to determine the concurrent validity of OASID. The CARS-2 is a screening tool for ASD in children, consisting of 15 items that can each be scored on a scale ranging from 1 point for normal behaviour to 4 points for severely abnormal behaviour. Half points can also be given, making it a 7-point Likert scale. A total raw score is calculated by adding scores on all 15 items. Higher scores indicate more severe symptoms of autism (Schopler et al. 2010). Reliability of the CARS-2 is reported to be satisfactory (McLellan 2014; O'Brien et al. 2001) and validity is moderate to good (Malcolm 2014).

The CARS-2 was chosen as an additional measure for validity within this study because of its psychometric properties. Additionally, it could easily be conducted using the tasks of OASID, without further burdening participants with additional testing.

### Procedure

Ethical approval for the study was gained from the Committee on Research Involving Human Subjects Arnhem-Nijmegen and conforms to World Medical Association declaration of Helsinki on the Ethical Principles for Medical Research Involving Human Subjects (World Medical Association 2013). As participants were deemed incapacitated, legal representatives of participants were asked for informed consent. Legal representatives were informed that the assessment would stop if there were reasons to believe the participant was unwilling to continue the assessment. This was never necessary.

Participants were tested within their own institution or school by one of three trained administrators. Assessments were done in quiet rooms with few if any stimuli to avoid distraction, with a familiar caregiver but without other clients present. Administration of OASID took between 20 and 55 min (36 min on average). After administration of OASID, caregivers were asked to fill out the PDD-MRS and provide record information regarding background, intellectual disability, and visual and auditory impairment.

The OASID assessment was recorded on video and was scored afterwards. The first author scored all videos. The second and third observers (two Master’s students in Educational and Pedagogical Science, both with experience with this target population and OASID assessments) scored 42 and 43 videos respectively, in order to assess inter-rater reliability. These second and third observer also scored 10 videos twice (after at least one month) to assess intra-rater reliability. Two additional Master’s students in Educational and Pedagogical Science administered the CARS-2 by observing the OASID video material.

Two experts in the field of ASD and intellectual disabilities and/or deafblindness independently observed videos of 14 randomly selected participants. One expert is a child psychiatrist with expertise in the field of deafblindness. The other expert has a PhD in special education and ample experience in diagnosing ASD in children with an intellectual disability. In addition to OASID video material, both experts received brief information from the participants’ records and a list of ASD criteria based on the DSM-5. The experts were blind to the protocol of OASID and method of scoring to prevent contamination between OASID scoring and their judgments. The expert judgments served as the gold standard for determining cut-off scores in this study. Because of the scope of the project and available funds we were able to have both experts observe 14 extra random cases. In addition to these 14 randomly selected participants from the current study, we added the 18 expert judgments from our previous study (de Vaan et al. 2016b), because these judgments were done in exactly the same manner by the same two experts, which enabled data pooling. As a result we had a larger number of expert judgments on which to base cut-off scores within the current study.

### Statistical Analyses

Intra- and inter-rater reliability was determined with weighted Cohen’s Kappa (Fleiss and Cohen 1973). Weighted kappa gives a more adequate estimation of reliability than unweighted kappa, since absolute differences between scores are taken into account. An intra-class correlation coefficient (ICC) with consistency in a two-way mixed model was used to assess reliability between three raters, as this corresponds best to weighted kappa (see Hallgren 2012). Reliability is deemed substantial when the kappa value is above 0.60, and almost perfect when it is above 0.80 (Landis and Koch 1977). ICC is good above 0.60 and excellent above 0.75 (see Barret 2001).

Internal consistency of scales was assessed with Cronbach’s alpha, with a required minimum of 0.7, but ideally near 0.9 (Kline 1993). However, in scales with a low number of items a lower Cronbach’s alpha is acceptable, as Cronbach’s alpha is very sensitive to number of items and will underestimate reliability when there are few items (Cortina 1993).

Concurrent validity was assessed by calculating Spearman-rank correlations between OASID scores and the PDD-MRS and CARS-2 scores as all these instruments aim to measure the same construct, which is ASD. In addition, expert judgments were also used to assess concurrent validity of OASID scores. Based on expert judgments the participants were split up into five groups: (1) both experts agreed on no ASD, (2) one expert was certain of no ASD, one expert doubted, (3) both experts doubted or they disagreed, (4) one expert was certain of ASD, one expert doubted, and (5) both experts agreed on the presence of ASD. To establish concurrent validity and cut-off scores, the 18 judgments from an earlier study (de Vaan et al. 2016b) were added to the 14 expert judgments in the current study.

## Results

### Intra-Rater Reliability

Two observers scored 10 videos they had scored before for a second time. The first observer had 89.3% exact agreement, corresponding to a weighted kappa of 0.89. The second observer had 89.4% exact agreement, corresponding to a weighted kappa of 0.90. According to the criteria of Landis and Koch (1977) the intra-rater reliability was almost perfect.

### Inter-Rater Reliability

All combinations of data from observers resulted in a weighted kappa of 0.63. The ICC over all three observers was also 0.63. The ICC for scale A ‘Social behaviour and communication’ was 0.64 and for scale B ‘Repetitive and Stereotyped behaviour’ 0.60. The subscale levels’ ICC are depicted in Table 1. According to the guidelines of Cicchetti (1994) these ICC’s are rated as ‘good’. The total and scale scores of OASID were highly correlated between all possible observer pairs; see Table 2.

**Table 1:** Reliabilities of scales and subscales of OASID

	Number of items	Cronbach’s Alpha	ICC
Scale A “Social Behavior and Communication”	21	0.91	0.64
Reciprocity	9	0.80	0.66
Non-verbal communication	3	0.59	0.65
Relationships	9	0.85	0.60
Scale B “Repetitive and Stereotyped Behavior”	19	0.85	0.60
Stereotyped and repetitive movements	7	0.73	0.70
Insistence on sameness	6	0.72	0.51
Restricted and fixated interests	3	0.43	0.60
Hyper- or hypo reactivity to sensory input	3	0.32	0.51
Total	40	0.94	0.63

**Table 2:** Correlations between OASID Scores

	Rater 1 and rater 2 (*n* = 43)	Rater 1 and rater 3 (*n* = 42)	Rater 2 and rater 3 (*n* = 42)
OASID total score	0.93*	0.92*	0.93*
Scale A “Social Behavior and Communication”	0.93*	0.90*	0.91*
Scale B “Repetitive and Stereotyped Behavior”	0.83*	0.82*	0.87*

*p < 0.01

### Internal Consistency

Cronbach’s alpha for scale A ‘social Behaviour and Communication’ was 0.91 and for scale B ‘Repetitive and Stereotyped Behaviour’ 0.85. These values can be interpreted as excellent and good, respectively (DeVellis 2012). The internal consistencies for the subscales are depicted in Table 1.

### Concurrent Validity

To assess concurrent validity, a one-tailed Spearman’s rank correlation was calculated between the total scores on OASID and the total scores on the PDD-MRS and the CARS-2. A small significant correlation was found between total scores on OASID and the PDD-MRS, *ρ* = .243, *p* = .038. The CARS-2 was rated by two observers. The total scores on OASID were moderately to highly significantly correlated with the total scores on the CARS-2 for both observers, *ρ* = .652, *p* < .001 and *ρ* = .801, *p* < .001, respectively.

A moderately strong Spearman-rank correlation was found between OASID scores and the ranks formed by the combined expert judgements regarding the presence of ASD, *ρ* = 0.67, *p* < 0.001.

### Cut-Off Scores

Most diagnostic and screening instruments for ASD provide one total score on which the final classification is based. Scores above the cut-off score are indicative of ASD. However, according to the definition of ASD in the DSM-5 (American Psychiatric Association 2013) this scoring method has two problems. Firstly, ASD occurs on a spectrum and is not merely a dichotomous label of ASD and no ASD. After one receives the diagnosis of ASD, that person can be classified within different levels of severity, to which different levels of required support correspond. Working with one cut-off score does not take into account these severity levels implicated in the DSM-5. Secondly, the DSM-5 clearly states that impairments in both domains of ASD must be present in order to diagnose ASD. A single total score could indicate impairments in both domains, but not necessarily, because it could also indicate the presence of many impairments in only one domain. In the latter case, when persons are diagnosed with ASD based on a high total score, they may not have all the required symptoms to assign the label ASD.

Because of these two problems with current scoring protocols, we propose a new protocol for scoring OASID, one that takes into account the spectrum of impairments in both domains. Firstly, a person must score high enough in both domains of ASD to receive an ASD classification (scale A ‘social behaviour and communication’ and scale B ‘repetitive and stereotyped behaviour’). Secondly, the resulting scores must contain information on ASD severity, namely more or fewer symptoms on the continuum of ASD.

The judgments by the two independent experts were used as the gold standard for determining cut-off scores. Cut-off scores were made for both scales separately. The experts reached exact agreement about the presence or absence of ASD in 13 cases. In 12 cases one expert doubted the presence of ASD and in 7 cases both experts doubted or they disagreed on the presence of ASD. For determining cut-off scores, only participants for whom the experts reached complete agreement were taken into account.

For both scales cut-off scores were determined by taking the highest scale score of the participant for whom there was consensus that ASD was not present and the lowest scale score of the participant with consensus on the presence of ASD. Therefore, both scales consist of two cut-off scores. Participants with scores below the lowest cut-off score were categorised as not showing symptoms of ASD on that scale. Scores between the two cut-off scores depicted mild symptoms of autism and scores above the highest cut-off score depicted true symptoms of autism on that scale. Table 3 shows the cut-off scores on both OASID scales and the corresponding classifications. As mentioned earlier, the DSM-5 states that impairments in both domains need to be present. In line with the DSM-5, a diagnosis of ASD can only be made when scores on OASID above the cut-off score on both scales are reached. Since ASD severity is distributed along a spectrum, the classifications of scores were also made according to this spectrum. Table 4 shows the classification of possible scores.

**Table 3:** Cut-off scores for OASID Scales

	Score on scale A ‘Social Behaviour and Communication	Score on scale B ‘Repetitive and Stereotyped Behaviour’
No autistic symptoms	11 and below	7 and below
Mild autistic symptoms	12–17	8–11
Severe autistic symptoms	18 and above	12 and above

**Table 4:** Severity of ASD symptoms

Score on OASID	Interpretation
No autistic symptoms on both scales	No ASD symptoms
No autistic symptoms on one scale, mild symptoms on other scale	No ASD symptoms
No autistic symptoms on one scale, severe symptoms on other scale	Mild ASD symptoms
Mild autistic symptoms on both scales	Mild ASD symptoms
Mild autistic symptoms on one scale, severe symptoms on other scale	Severe ASD symptoms
Severe autistic symptoms on both scales	Profound ASD symptoms

OASID comprises two scales, which are (A) Social behaviour and communication, and (B) stereotyped and repetitive behaviours. Symptoms of ASD must be present on both scales in order to diagnose ASD. To interpret the severity of ASD, an interpretation of symptom severity is required; this can be derived from Table 3

### Potentially Confounding Factors

Afterwards possible confounding factors for the ASD classification were checked**.** No correlation between age and OASID score was found, *r* = −.089, *p* = .51, suggesting that OASID scores are most likely unrelated to and not confounded by age.

Chi square tests were performed to assess the proportion of the different levels of visual impairments, auditory impairments and intellectual disabilities among the four proposed groups of ASD (see paragraph cut-off scores). Three visual impairment groups were made: (1) visually impaired but uses sight, (2) blind with light perception and (3) blind without light perception. Chi square tests revealed that levels of visual impairments were not associated with ASD groups, *χ*^2^ (6, *n* = 60) = 7.87, *p* = .25. Three auditory impairment groups were made: (1) no auditory impairment, (2) auditory impairment and (3) deaf. Level of auditory impairment was not associated with ASD groups, *χ*^2^ (6, *n* = 60) = 8.48, *p* = .21. For level of intellectual disability three groups were made: (1) moderate intellectual disability, (2) severe intellectual disability and (3) profound intellectual disability. Level of intellectual disability was associated with ASD groups, *χ*^2^ (6, *n* = 60) = 23.27, *p* = .001. Persons with profound intellectual disability were more often classified with profound ASD (60%) than people with a severe intellectual disability (12, 5%). No one with a moderate intellectual disability was in this ASD group.

## Discussion

Because it is difficult to assess the presence of ASD in people with combined sensory and intellectual disabilities, OASID was developed to assist in this process. The current study tested the reliability and validity of OASID on a group of participants with a moderate to profound intellectual disability combined with a visual impairment or deafblindness. This study elaborated on a previous study which described the psychometric properties of OASID for a relatively small sample of 18 participants (de Vaan et al. 2016b). The results of the current study with 60 participants showed excellent intra-rater reliability, good inter-rater reliability, good internal consistency of scales and good concurrent validity of OASID with two other instruments and expert judgement. On subscale level, reliability was low only for the subscales with few items (i.e. 3 items), namely ‘non-verbal communication’, ‘restricted and fixated interests’ and ‘hyper- or hypo reactivity to sensory input’. This does not necessarily mean that the scales are not reliable, since Cronbach’s alpha underestimates reliability when there are a small number of items (Cortina 1993). For clinical interpretations of individual OASID scores, we recommend the use of only the scale scores and total scores, not the scores on discrete subscales.

To establish concurrent validity, correlations were calculated between OASID scores and scores on the PDD-MRS, CARS-2 and expert judgments. A moderately strong correlation was found between OASID scores and expert judgments. Partly, the experts based their judgments on video material of the OASID assessment, but they had no insight into how OASID was scored; they only watcged play sessions. Therefore, we believe contamination between OASID and the expert’s opinion is kept to a minimum. We found significant correlations between OASID scores and the PDD-MRS and the CARS-2 scores, which points to good concurrent validity. This was expected because both instruments were also developed to assess the presence of ASD. The correlation between the OASID and both expert judgments and the CARS-2 scores were moderately strong to strong. This is in contrast to the correlation with the PDD-MRS, which was significant but also small.

The relatively high correlation between OASID, the expert judgments and the CARS-2 scores may partly be due to the fact that they were all based on the same video recordings. However, this also means that contextual factures are the same and cannot be responsible for variation in outcome of the different instruments. The PDD-MRS was chosen as a measure of concurrent validity because it is one of the few instruments available that is specifically developed for assessing ASD in people with intellectual disabilities (Kraijer and de Bildt 2005). Nevertheless, the PDD-MRS was not developed for use in people with additional sensory impairments, hence explaining the rather low correlation with OASID. To estimate the severity of ASD symptoms, OASID is probably a better fit for people with sensory and intellectual disabilities than the CARS-2 and PDD-MRS.

In the second part of this study, a heuristic was proposed for scoring OASID. The DSM-5 (American Psychiatric Association 2013) states that ASD consists of two behavioural domains and that impairments in both domains need to be present before a classification of ASD can be established. It further acknowledges that when someone has ASD, the behaviours occur on a severity spectrum, leading to different levels of ASD instead of only a strict distinction between ASD and no ASD. Diagnostic instruments should therefore take these severity levels into account (Mehling and Tassé 2015). Furthermore, behaviours symptomatic of autism need to be scored in both behavioural domains described in the DSM-5. When diagnosing ASD with a total score alone, a high score does not necessarily mean that impairments occur in both domains. In contrast to most of the existing diagnostic instruments, the advantage of our heuristic in establishing ASD severity is that it takes into account the score in both ASD domains and distinguishes between different levels of ASD severity. The latter resulted in four possible levels of severity: no, mild, severe and profound ASD symptoms.

The current study focused on a rather broad age range within our target population. Both children and adults were taken into account in the development of OASID. This is in contrast to most studies, where ASD instruments are usually aimed at children alone or have separate norms for children and adults (de Vaan et al. 2016a). However, since all participants have a moderate to profound intellectual disability and impaired communicative abilities, we do believe they can be grouped together for the purpose of the current study. This belief was strengthened by a lack of a correlation between age and OASID scores. Level of visual impairment and level of auditory impairment were also taken into account as possible confounders. However, chi-square tests indicated that level of visual and auditory impairments were equally distributed across ASD groups; hence we do not believe these impairments severely affected OASID scores. Level of intellectual disability, however, was associated with ASD group classification. Specifically, compared to the other ASD groups, in the group with profound ASD symptoms, a high proportion of people with profound intellectual disabilities were found. This is not uncommon since there is ample evidence that the prevalence of ASD is higher in people with intellectual disabilities than in people without, and that severity of intellectual disability and severity of ASD are related (Matson and Shoemaker 2009; O’Brien and Pearson 2004; de Bildt et al. 2005). The current results are in line with these findings. This study was based on the results of 60 participants, making the use of subgroups based on disabilities or age statistically difficult. For future research, and for the further development of OASID, it is recommended that OASID is tested on larger groups of participants, to fully study the effects of age, level of intellectual disability and level of sensory impairments. Only after these studies would it be possible to determine if different cut-off points are required for specific subpopulations.

A possible limitation of the current study is the gold standard used for determining the cut-off scores, namely using consensus judgements of two experts who used video material and brief anamnestic information on the participants. Though the ADOS (Lord et al. 1999) and ADI-R (Rutter et al. 2003) are often seen as a gold standard in ASD diagnoses (de Bildt et al. 2004; Reaven et al. 2008), the ADOS was found over-inclusive in individuals with an intellectual disability and many tasks could not be assessed in individuals with severe disabilities (Sappok et al. 2013). The ADI-R is only found suitable for people with a developmental age of above two years (Rutter et al. 2003). Because the ADOS and ADI-R are not suitable for our population, we chose to use expert judgments in addition to two other instruments that we felt came closest to being valid instruments for our population. We readily admit that the Autism Behavior Checklist (ABC) as used by Dammeyer (2014) could have been an alternative, but administering this checklist did not fit our study plan. Moreover, although the ABC was validated on people with intellectual disabilities and deafblindness, it is also not validated for people with a combination of these disabilities. Since up to now no valid instruments exist to diagnose ASD in people with combined sensory and intellectual disabilities (de Vaan et al. 2016a), it was not possible to use results of other instruments as the gold standard. Where no gold standard exists, expert consensus is a commonly used method.

Unfortunately, in many participants no consensus was reached because either one or both of the experts doubted the presence of ASD. This could be partly caused by the limited information they received about participants. A formal diagnosis of ASD can only be made by a multidisciplinary team in a multimethod assessment procedure combined with anamnestic information (Risi et al. 2006; Rutter 2006; Volkmar et al. 2014). In addition, the experts’ classifications were based on their final decision concerning the presence of ASD, not on their judgments on individual behavioural characteristics or the severity of symptoms. Future research could focus on the criteria that experts use to come to their decisions regarding the presence of ASD, and also on designing guidelines for best practices to come to a clinical diagnosis of ASD.

In conclusion, OASID proved to be a reliable and valid tool that scores ASD in line with DSM-5 criteria, expert judgments and scores on two instruments. It was developed specifically for people with combined intellectual disabilities and sensory impairments, aimed at overcoming the risk of over- diagnosing ASD in this group. The current study elaborated on a pilot study with a larger sample of 60 participants. The results of this study indicate that OASID can be a useful tool in assessing behaviour of individuals with combined sensory and intellectual disabilities. Reference points were established in order to interpret the severity of ASD symptoms, which adds to the clinical usability of OASID.
